# Supplementary material for: Non-linear relationships between daily temperature extremes and US agricultural yields uncovered by global gridded meteorological datasets
Source: Nat Commun. 2024 May 31;15:4638. doi: 10.1038/s41467-024-48388-w (PMC11143199; doi:10.1038/s41467-024-48388-w)
Supplement: Supplementary file 1 — Supplementary Information [file 41467_2024_48388_MOESM1_ESM.pdf]

Supplementary Information

Non-linear relationships between daily temperature extremes and US agricultural yields uncovered by global gridded meteorological datasets

Dylan Hogan and Wolfram Schlenker

Supplementary Note 1: Contrasting Spatial and Temporal Resolution

There are two differences between ERA5-Land and PRISM. The former is spatially more aggregated (11km grid versus 4km grid size), yet temporarily less aggregated (24 hourly temperature observations rather than providing the daily maximum and minimum temperature). To further examine the effect of spatial aggregation, we aggregate the raw temperature data from the finer scale to the more aggregate scale and then derive again our non-linear temperature transformation. To further examine the effect of temporal aggregation, we aggregate the hourly data to the daily level.

In the case of the spatially disaggregated PRISM data, we first aggregate minimum and maximum temperature on the 4km PRISM grid to the same 11km resolution of the ERA5-Land grid and then re-derive the temperature measures (degree-days and precipitation). We focus on the piecewise linear model in Figure 2, the most parsimonious model having only four weather variables: two degree days variables as well as a quadratic in precipitation, that does best in explaining corn yields. Similarly, for the temporally more disaggregated ERA5-Land weather data, we take the minimum and maximum of the 24 hourly observations and

then use the sine-interpolation between minimum and maximum temperature (see Methods).

The results are shown in Supplementary Figure 1. Note that aggregating the PRISM data to the same spatial resolution as the ERA5-Land grid (second bar) is in between the height of the PRISM grid (first bar) and ERA5-Land bar (third bar), i.e., about half of the difference in the better out-of-sample prediction of PRISM is attributable to a finer scale that better captures local extremes. On the other hand, the finer temporal resolution of the ERA-5 has no benefit—when we aggregate the hourly data to the daily minimum and maximum (fourth bar), it is statistically indistinguishable from using the hourly data (third bar).

Supplementary Figure 1: Out-of-Sample Model Prediction Accuracy: Spatial and Temporal Resolution

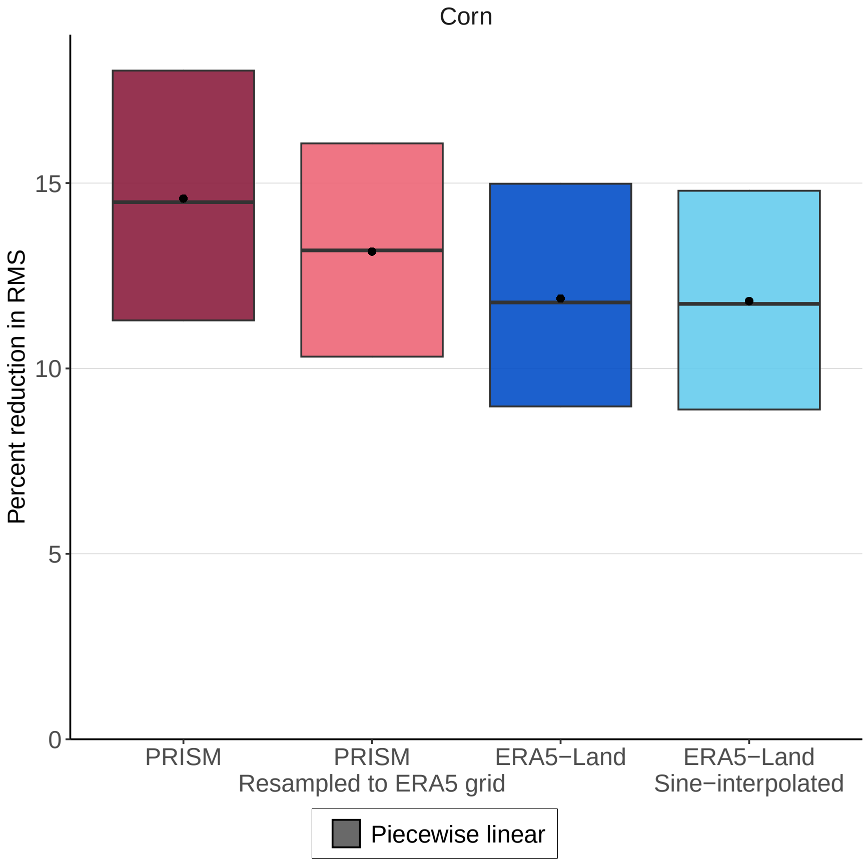

Notes: Figure compares out-of-sample corn yield predictions for piecewise linear regression models estimated using weather observations from PRISM and ERA5-Land data sets. Red and blue (first and third) bars show the percent reduction in root-mean-squared error (RMS) relative to a baseline model as shown in Figure 2. The pink (second) bar shows results for a piecewise linear model estimated on PRISM data aggregated from a 1/24 degree grid to a 0.1 degree grid. The cyan (fourth) bar shows a model estimated on degree days calculated using a sinusoidal interpolation of daily maximum and minimum temperature values, rather than from hourly observations in the raw data. For each data set, piecewise linear response functions are estimated 1,000 times, each time randomly sampling 85% of the years from the full panel. RMS is calculated based on each piecewise linear model's prediction of the remaining 15% of years. Boxes, horizontal lines, and points represent the interquartile range, median, and mean of RMS reductions from the 1,000 draws, respectively.

Supplementary Note 2: Precipitation Response Functions

Supplementary Figure 2 compares the effect of rainfall on yields across PRISM, ERA5-Land, and GMFD. Response functions from each climate data set and crop are concave. For corn, PRISM and GMFD estimate statistically similar response functions, with ERA5-Land estimating smaller effects. For soybeans, all three data sets produce similar responses.

Supplementary Figure 2: Precipitation Response Functions for Corn and Soybeans Yields

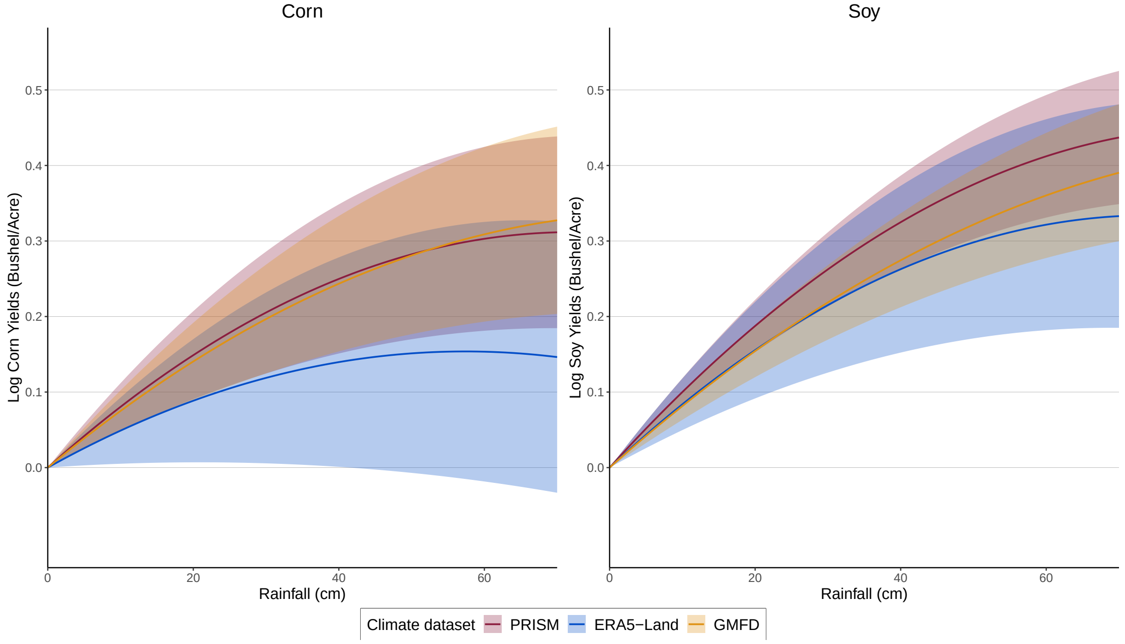

Notes: Each graph estimates the relationship between US yields and precipitation using both the fine-scaled PRISM data set (shown in red) as well as the more aggregate but globally available ERA5-Land (shown in blue) and GMFD (shown in yellow). Lines indicate estimated response functions from the panel regression models described in Methods and bands indicate the 95% confidence intervals. Precipitation response functions are quadratic functions of total rainfall over the growing season (March - September). The left panel shows relationships for corn yields and the right panel shows relationships for soybean yields. Response functions represent the percent change in yields associated with a change in rainfall relative to a year with zero rainfall over the growing season. Regressions control for a piecewise linear function of temperature, county fixed effects, and state-specific quadratic time trends (see Methods).

Supplementary Note 3: County-level Yield Projections from 2°C warming

Supplementary Figure 3 provides county-level maps of predicted changes in crop yields under a uniform 2°C of warming for each crop-climate data set combination. These are the changes relative to a baseline of no warming/ Maps show the central estimate of climate change impacts from the piecewise linear specification. Some counties in the northern and central US experience yield benefits from 2°C warming; however, aggregate impacts across crops and warming scenarios are negative and driven by increases in the number of extremely hot days. For corn yields, county-level yield projections differ by 2.5% on average (inter-quartile range IQR = $[0.9, 3.6]$) when the ERA5-Land is used. Under GMFD the average is slightly larger at 2.9% (IQR = $[1.6, 4.0]$). For soybean yields, county-level yield projections differ by 2.8% on average (IQR = $[0.9, 4.1]$) for ERA5-Land and 2.1% on average (IQR = $[1.0, 3.0]$) for GMFD. The difference across weather data sets is hence much smaller than the difference across space.

Supplementary Figure 3: Projected County Level Corn and Soybean Yield Impacts from 2°C Warming

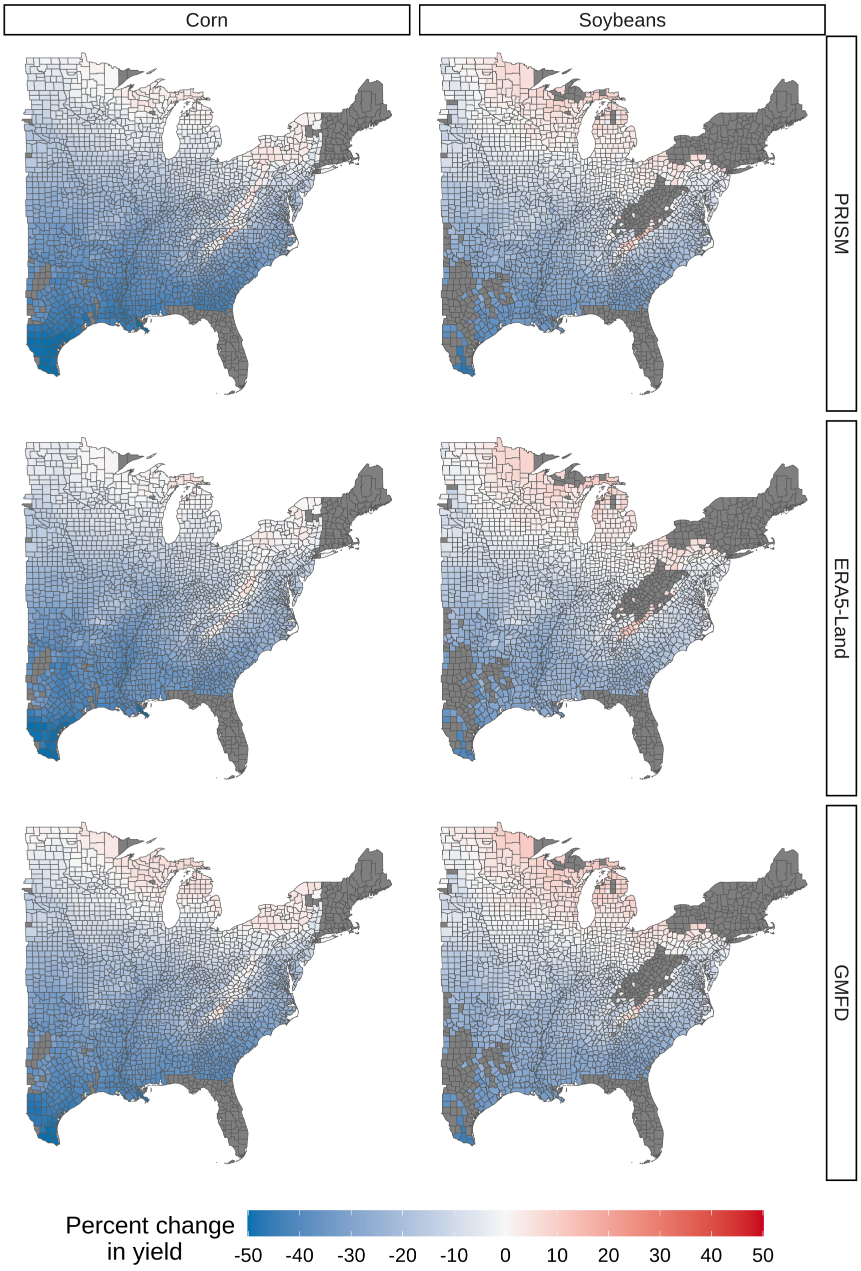

Notes: Figure includes maps of the US east of the 100th meridian. Maps depict the county-level effect of a 2°C uniform warming scenario on crop yields, as predicted by piecewise linear relationships between weather and crop yields (see Methods). The two columns of maps represent corn and soybeans yields, and the three rows represent the three climate data sets, PRISM, ERA5-Land, and GMFD. Values represent the percent change in crop yields, where blue represents yield losses due to warming and red represents yield benefits. Maps are generated by the authors using shapefiles obtained from the US Census Bureau: `https://www.census.gov/geographies/mapping-files/time-series/geo/tiger-line-file.html`.

Supplementary Note 4: Robustness of Piecewise-linear Model

Supplementary Datasets 1 and 2 provide regression coefficients for the most parsimonious model of US corn and soybean yields, respectively: a piecewise linear function of temperature and a quadratic function of total precipitation over the growing season. The tables consist of three panels, each corresponding to a different climate data set (PRISM, ERA5-Land, and GMFD, respectively). All columns include county fixed effects. Columns 1-3 show results from models with state-specific quadratic time trends, and columns 4-6 show results with year fixed effects. Within these groups, the columns account differently for correlation of errors. Columns 1 and 4 cluster the standard errors at the state level. Note that column 1 is the preferred specification, in terms of fixed effects and standard errors, presented throughout the main paper. Columns 2 and 5 show results with standard errors clustered at the year level. Columns 3 and 6 show results based upon Conley standard errors, which adjust for spatial correlation of errors 1000 km around each county[1].

Supplementary Note 5: Climate data comparison

Supplementary Figures 4 and 5 provide summaries of temperature data for PRISM, ERA5-Land, and GMFD. In particular, Supplementary Figure 4 shows pair-wise comparisons of degree days above 30°C. Temperature values shown in each panel are residuals from a regression of degree days on county fixed effects and state-level quadratic time trends. Thus, values are demeaned at the county level and flexible trends are removed from each temperature record. Black points represent the residual county-year temperature values and blue lines represent trends. Partial correlations, i.e., correlations in the residuals, are provided on each panel. Residual temperature variation is highly correlated across climate datasets, with PRISM and ERA5-Land showing the highest correlation of about 0.92. PRISM and GMFD are slightly less correlated (0.9) and the two global data sets are least correlated (0.86). Figure 5 provides maps of county-level temperature over the growing season, averaged over the sample. From top to bottom, maps represent temperature measurements calculated from PRISM, ERA5-Land, and GMFD. The left column shows degree days above 30°C and the right column shows average temperature.

Supplementary Figure 4: Partial correlation of temperature observations across data sets

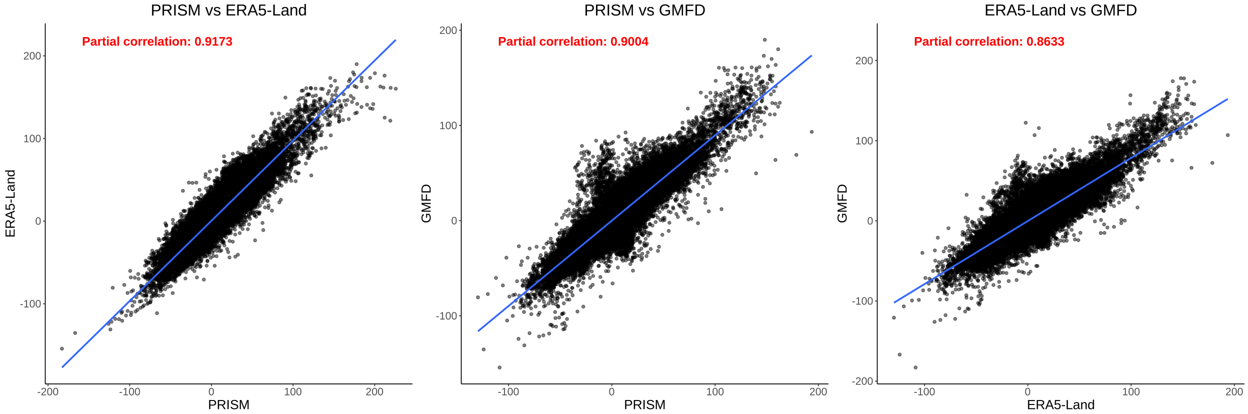

Notes: Figure provides scatter plots of temperature observations from PRISM, ERA5-Land, and GMFD. Temperature is measured as total degree days above 30°C in a growing season. Temperature values are the residuals of a regression of degree day observations from each data set on county fixed effects and quadratic state-specific time trends. Thus, values are demeaned at the county level and quadratic state-specific trends are removed from each temperature record. Blue lines represent linear trend lines. The Pearson correlation coefficient of the demeaned and detrended temperature values is provided in each panel in red.

Supplementary Figure 5: Average county-level heat exposure over the growing season

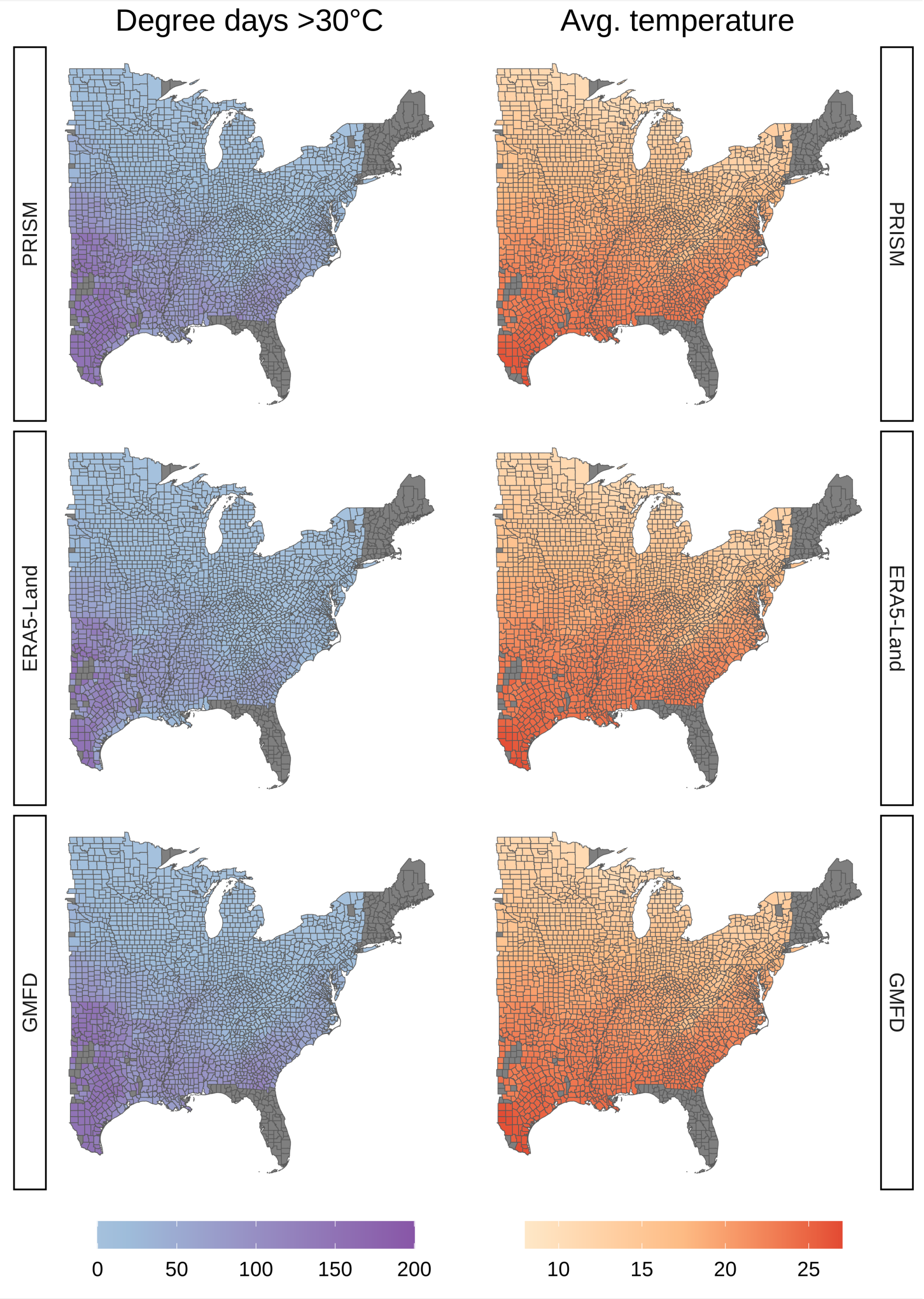

Notes: Figure includes maps of the US east of the 100th meridian depicting two measures of county-level heat exposure. The left column of maps provides the number degree days above 30°C during the growing season (March-September) averaged over our sample (1950-2019 for PRISM and ERA5-Land and 1950-2010 for GMFD). The right column shows county-level average temerature over the growing season, again averaged over the sample. From top to bottom, maps represent temperature measurements calculated from PRISM, ERA5-Land, and GMFD. Maps are generated by the authors using shapefiles obtained from the US Census Bureau: `https://www.census.gov/geographies/mapping-files/time-series/geo/tiger-line-file.html`.

References

[1] Conley, T. G. GMM estimation with cross sectional dependence. *Journal of econometrics* **92**, 1–45 (1999).
