# Supplementary material for: Non-linear relationships between daily temperature extremes and US agricultural yields uncovered by global gridded meteorological datasets
Source: Nat Commun. 2024 May 31;15:4638. doi: 10.1038/s41467-024-48388-w (PMC11143199; doi:10.1038/s41467-024-48388-w)
Supplement: Supplementary file 2 — Reporting Summary [file 41467_2024_48388_MOESM2_ESM.pdf]

nature portfolio

Corresponding author(s): Dylan Hogan

Last updated by author(s): Apr 15, 2024

# Reporting Summary

Nature Portfolio wishes to improve the reproducibility of the work that we publish. This form provides structure for consistency and transparency in reporting. For further information on Nature Portfolio policies, see our Editorial Policies and the Editorial Policy Checklist.

## Statistics

For all statistical analyses, confirm that the following items are present in the figure legend, table legend, main text, or Methods section.

| n/a | Confirmed | |
|---|---|---|
| ☐ | ☒ | The exact sample size (*n*) for each experimental group/condition, given as a discrete number and unit of measurement |
| ☐ | ☒ | A statement on whether measurements were taken from distinct samples or whether the same sample was measured repeatedly |
| ☐ | ☒ | The statistical test(s) used AND whether they are one- or two-sided<br>*Only common tests should be described solely by name; describe more complex techniques in the Methods section.* |
| ☐ | ☒ | A description of all covariates tested |
| ☐ | ☒ | A description of any assumptions or corrections, such as tests of normality and adjustment for multiple comparisons |
| ☐ | ☒ | A full description of the statistical parameters including central tendency (e.g. means) or other basic estimates (e.g. regression coefficient) AND variation (e.g. standard deviation) or associated estimates of uncertainty (e.g. confidence intervals) |
| ☐ | ☒ | For null hypothesis testing, the test statistic (e.g. *F*, *t*, *r*) with confidence intervals, effect sizes, degrees of freedom and *P* value noted<br>*Give P values as exact values whenever suitable.* |
| ☒ | ☐ | For Bayesian analysis, information on the choice of priors and Markov chain Monte Carlo settings |
| ☒ | ☐ | For hierarchical and complex designs, identification of the appropriate level for tests and full reporting of outcomes |
| ☒ | ☐ | Estimates of effect sizes (e.g. Cohen's *d*, Pearson's *r*), indicating how they were calculated |

*Our web collection on statistics for biologists contains articles on many of the points above.*

## Software and code

Policy information about availability of computer code

| | |
|---|---|
| Data collection | No software was used for data collection. |
| Data analysis | Climate data aggregation and cleaning was performed using Python (version 3.11.6) and the statistical analysis was performed using the R statistical package (version 4.0.2). The code developed to produce the data cleaning and analysis described in this paper and the Supplementary Information is available in the following repository: https://github.com/dylanhogan/climate_data_yields under a GNU General Public License v3.0 |

For manuscripts utilizing custom algorithms or software that are central to the research but not yet described in published literature, software must be made available to editors and reviewers. We strongly encourage code deposition in a community repository (e.g. GitHub). See the Nature Portfolio guidelines for submitting code & software for further information.

## Data

Policy information about availability of data

All manuscripts must include a data availability statement. This statement should provide the following information, where applicable:

- Accession codes, unique identifiers, or web links for publicly available datasets
- A description of any restrictions on data availability
- For clinical datasets or third party data, please ensure that the statement adheres to our policy

The analysis relies on public use weather data from PRISM (https://prism.oregonstate.edu/), ERA5-Land (https://cds.climate.copernicus.eu/) and GMFD (https://rda.ucar.edu/datasets/ds314.0/), as well as data on agricultural production from U.S. Department of Agriculture's National Agricultural Statistical Service (https://

www.nass.usda.gov/Data_and_Statistics/). We also rely on cropland data layers from USDA (https://www.nass.usda.gov/Research_and_Science/Cropland/SARS1a.php) and FAO GAEZ (https://gaez.fao.org/) as well as weather station locations from National Climate Data Center (https://www.ncei.noaa.gov/cdo-web/). Spatial aggregation of climate data and maps produced in the Supplementary Information rely on shapefiles provided by the US Census Bureau (https://www.census.gov/geographies/mapping-files/time-series/geo/tiger-line-file.html). The cleaned and aggregated data used to produce the analysis in the paper and the Supplementary Information are available in the following repository: https://doi.org/10.7910/DVN/XRMDBW under a Creative Commons 4.0 license.

## Research involving human participants, their data, or biological material

Policy information about studies with human participants or human data. See also policy information about sex, gender (identity/presentation), and sexual orientation and race, ethnicity and racism.

| | |
|---|---|
| Reporting on sex and gender | N/A |
| Reporting on race, ethnicity, or other socially relevant groupings | N/A |
| Population characteristics | N/A |
| Recruitment | N/A |
| Ethics oversight | N/A |

Note that full information on the approval of the study protocol must also be provided in the manuscript.

# Field-specific reporting

Please select the one below that is the best fit for your research. If you are not sure, read the appropriate sections before making your selection.

☐ Life sciences ☒ Behavioural & social sciences ☐ Ecological, evolutionary & environmental sciences

For a reference copy of the document with all sections, see nature.com/documents/nr-reporting-summary-flat.pdf

# Behavioural & social sciences study design

All studies must disclose on these points even when the disclosure is negative.

| | |
|---|---|
| Study description | This study assesses whether two globally representative weather datasets uncover similar quantitative relationships with agricultural yields as existing data sets used in the literature to estimate climate impacts. |
| Research sample | Our research sample is counties in the United States east of the 100th meridian and, in a second step, all countries in Sub-Saharan Africa. The US sample was chosen because of the availability of high quality data on agricultural yields and weather with which to compare the global data sets that are the focus of our analysis. We use data for counties east of the 100th meridian to avoid the confounding effect of irrigation, which is prevalent in the western US. We then focus on Sub-Saharan Africa to evaluate the data sets in a relatively data sparse region of the world. Instead of agricultural census data, we use satellite proxies for yields in this region. |
| Sampling strategy | For the US, we compile a data set containing all available agricultural census data from USDA. This includes all census data for counties east of the 100th meridian for the years between 1950 and 2020. For the Sub-Saharan Africa analysis, we perform a stratified random sample of grid cells from the weather grid and vegetation index to evaluate the importance of in-situ weather stations for predicting yields. In particular, since (1) there are many more grid cells without stations than with stations in our sample, and (2) weather stations are not randomly distributed throughout Sub-Saharan Africa, we perform a stratified random sample so that relationships estimated in locations without weather stations have a similar country composition as relationships estimated in locations with nearby weather stations. The stratified random sample of grid cells without weather stations is then compared to the universe of grid cells with weather stations, where the sample sizes are about equal. |
| Data collection | All data used for the analysis is publicly available online. Therefore, no instruments or devices were used and we did not have study participants. |
| Timing | This study does not involve primary data collection. |
| Data exclusions | No data were excluded from the analysis. |
| Non-participation | This study does not involve participants. |
| Randomization | No randomization was performed for this study. |

# Reporting for specific materials, systems and methods

We require information from authors about some types of materials, experimental systems and methods used in many studies. Here, indicate whether each material, system or method listed is relevant to your study. If you are not sure if a list item applies to your research, read the appropriate section before selecting a response.

## Materials & experimental systems

| n/a | Involved in the study |
|---|---|
| ☒ | ☐ Antibodies |
| ☒ | ☐ Eukaryotic cell lines |
| ☒ | ☐ Palaeontology and archaeology |
| ☒ | ☐ Animals and other organisms |
| ☒ | ☐ Clinical data |
| ☒ | ☐ Dual use research of concern |
| ☒ | ☐ Plants |

## Methods

| n/a | Involved in the study |
|---|---|
| ☒ | ☐ ChIP-seq |
| ☒ | ☐ Flow cytometry |
| ☒ | ☐ MRI-based neuroimaging |

# Plants

| | |
|---|---|
| Seed stocks | *Report on the source of all seed stocks or other plant material used. If applicable, state the seed stock centre and catalogue number. If plant specimens were collected from the field, describe the collection location, date and sampling procedures.* |
| Novel plant genotypes | *Describe the methods by which all novel plant genotypes were produced. This includes those generated by transgenic approaches, gene editing, chemical/radiation-based mutagenesis and hybridization. For transgenic lines, describe the transformation method, the number of independent lines analyzed and the generation upon which experiments were performed. For gene-edited lines, describe the editor used, the endogenous sequence targeted for editing, the targeting guide RNA sequence (if applicable) and how the editor was applied.* |
| Authentication | *Describe any authentication procedures for each seed stock used or novel genotype generated. Describe any experiments used to assess the effect of a mutation and, where applicable, how potential secondary effects (e.g. second site T-DNA insertions, mosiacism, off-target gene editing) were examined.* |
