# Supplementary material for: Non-linear relationships between daily temperature extremes and US agricultural yields uncovered by global gridded meteorological datasets
Source: Nat Commun. 2024 May 31;15:4638. doi: 10.1038/s41467-024-48388-w (PMC11143199; doi:10.1038/s41467-024-48388-w)
Supplement: Supplementary file 4 — Description of Additional Supplementary Files [file 41467_2024_48388_MOESM4_ESM.pdf]

File Name: Supplementary Data 1
Description: Table shows estimated coefficients from the piecwise linear panel regression models on corn yields in the US (see Methods). Standard errors are provided in parentheses. Columns show different assumptions regarding controls for temporal trends and levels of correlation in the calculation of standard errors. Significance levels of the coefficients are marked as: ***: P < 0.001, **: P<0.01, *: P<0.05., .:P<0.1. Degree day and linear precipitation terms scaled by 10^3. Quadratic precipitation term scaled by 10^6.

File Name: Supplementary Data 2
Description: Table shows estimated coefficients from the piecwise linear panel regression models on soybean yields in the US (see Methods). Standard errors are provided in parentheses. Columns show different assumptions regarding controls for temporal trends and levels of correlation in the calculation of standard errors. Significance levels of the coefficients are marked as: ***: P < 0.001, **: P<0.01, *: P<0.05., .:P<0.1. Degree day and linear precipitation terms scaled by 10^3. Quadratic precipitation term scaled by 10^6.
